# Experimental Study on Crack Evolution Characteristics in Guar Gum-Modified Silty Clay

**DOI:** 10.3390/polym17131841

**Published:** 2025-06-30

**Authors:** Xiyan Jiang, Wanxin Hou, Dongning Zhang, Zhibao Guo, Dameng Wang, Xu Wang

**Affiliations:** 1 College of Civil Engineering, Hebei University of Architecture, Zhangjiakou 075000, China; 2 Hebei Provincial Engineering Technology Innovation Center for Transportation Infrastructure in Cold Regions, Zhangjiakou 075000, China

**Keywords:** guar gum-improved soil, crack parameters, fractal dimensions, probability entropy

## Abstract

The formation of soil cracks in soil slopes can compromise structural integrity. Guar gum, as a natural high-molecular-weight biopolymer, offers environmental and economic advantages in soil stabilizers due to its biodegradability, strong binding properties, and ability to form a three-dimensional network structure. To investigate its improvement effects, outdoor dry shrinkage cracking tests were conducted on silt loam using different guar gum dosages. Image preprocessing was performed using Photoshop software, and Python algorithms combined with the PCAS system were employed to quantitatively analyze the development process of cracks, revealing the evolution patterns of basic crack parameters, fractal dimensions, and probability entropy. The results indicate the following: (1) the addition of guar gum improves the water retention capacity of the soil, with the average moisture content of the samples decreasing as the guar gum content increases; (2) as the guar gum content increased, the total length, total area, and surface crack ratio of the cracks all increased, but the average crack width decreased significantly, with the maximum decrease reaching 9.8%, indicating that guar gum can effectively suppress the expansion of crack width and slow down the infiltration rate of rainwater; (3) the fractal dimension of crack area is less affected by guar gum content, while the fractal dimension of crack length is significantly influenced by guar gum content. Combining both parameters can effectively characterize crack morphology and distribution. The final fractal dimension of crack length generally ranges from 1.2 to 1.3, while the fractal dimension of the crack area remains stable between 1.55 and 1.65; (4) the addition of guar gum has a minor effect on the probability entropy of cracks, with a change of less than 3%, indicating that it does not significantly influence the randomness of cracks. Therefore, this study confirms that guar gum has a significant effect in controlling crack width and optimizing the uniformity of the crack network. Through its mechanisms of binding soil particles and delaying drying shrinkage, it provides an important reference for the ecological protection of cohesive soil slopes.

## 1. Introduction

Clayey soils are widely distributed in China, and clayey soils in their naturally deposited state have high density [1]. Large-scale engineering construction causes damage to soil structure, leading to the gradual loosening of cohesive soil slopes and inducing cracks. Cracks, as an inherent characteristic of fine-grained soils [2], are caused by the expansion and contraction of cohesive soil minerals [3]. The appearance of cracks disrupts the integrity of the soil, providing a pathway for surface water infiltration, which in turn causes the cracks to widen continuously and ultimately leads to the instability of the soil slope [4].

Drying shrinkage cracking of cohesive soils is a common phenomenon in nature. This phenomenon weakens the engineering properties of cohesive soils and directly triggers many geological and geotechnical engineering problems. Domestic and international scholars have studied the drying shrinkage cracking of cohesive soils and carried out soil reinforcement and improvement measures. Zhang et al. [5] investigated the effect of biochar on the drying shrinkage cracking characteristics of clay and conducted an in-depth analysis of its mechanism. Ding Xuan Ming et al. [6] investigated the effect of PVA fibers on the properties of silt, finding that they effectively improved the overall performance of the soil, significantly inhibiting soil shrinkage and crack propagation. Wang Feng [7] et al. conducted experiments on the indoor dry shrinkage cracking characteristics of clay using basalt fiber. The results showed that it could effectively reduce the crack width and ratio. Xie et al. [8] used nylon fibers and enzyme-based products to improve expanded clay, finding that it could significantly alter the crack development pattern, thereby mitigating the impact of cracks. Yuan et al. [9] conducted indoor experiments to study the drying cracking characteristics of saline-alkali soil with different polypropylene fiber contents. They found that polypropylene fibers can significantly improve the structural stability and integrity of the soil, effectively inhibiting the formation and expansion of cracks.

Regarding research on biopolymers and new modification technologies, the academic community is focusing on the interaction mechanisms between materials and soil particles. Liu et al. [10] used industrial waste materials such as bottom ash as admixtures to improve soil properties and found that they could slow down the formation of cracks to a certain extent. Liu et al. [11] investigated the effect of microbial-induced calcite precipitation (MICP) on clay cracking. The results showed that as the number of MICP treatments increased, the degree of cracking on the soil surface decreased significantly. Qi et al. [12] investigated the effect of polyacrylamide and polypropylene mixtures on the drying cracking of clay. They found that adding polyacrylamide slightly reduced the cracking and fragmentation of the soil. In contrast, adding polypropylene slightly increased the cracking and fragmentation of the soil. Chang et al. [13,14,15] separately studied the improvement of sandy soil and clay by biopolymers. The experimental results showed that biopolymers can aggregate fine soil particles into aggregates through hydrogen bonds and electrostatic bonds, thereby reducing soil particle pores, enhancing cohesion, and reducing cracks. Ouyang Miao et al. [16] conducted crack evolution experiments on expanded clay modified with xanthan gum biopolymer. The aforementioned studies indicate that both fibers and biopolymers can serve as environmentally friendly soil amendment materials, while research on the use of biopolymers for soil amendment remains relatively limited.

Among numerous biopolymers, guar gum has emerged as a promising research direction due to its unique advantages. Its wide availability, cost-effectiveness, and environmental friendliness have attracted significant attention from the academic community. Current research primarily focuses on investigating the effects of guar gum on soil mechanical properties, such as enhancing cohesion and interparticle friction angle [17].

Guar gum, as a high-molecular-weight biopolymer, can suppress drying shrinkage cracking by enhancing the overall integrity and deformation resistance of soil. However, few studies have focused on improving the drying shrinkage cracking characteristics of soil. Different guar gum biopolymers were selected to modify the soil, and outdoor drying shrinkage cracking tests were conducted, with bare soil serving as the control group. The moisture content of the samples was monitored throughout the process. Using image processing technology, basic parameters of cracks, fractal dimension, crack development rate, and changes in crack probability entropy were obtained. A dynamic evolution mechanism analysis of cracks was conducted, leading to the identification of dynamic evolution patterns of cracks under different guar gum content levels and the reinforcement effects of guar gum. This provides a theoretical basis for further optimizing ecological protection methods for clay slopes.

## 2. Materials and Methods

### 2.1. Test Materials

#### 2.1.1. Soil Samples

**Figure 1:**
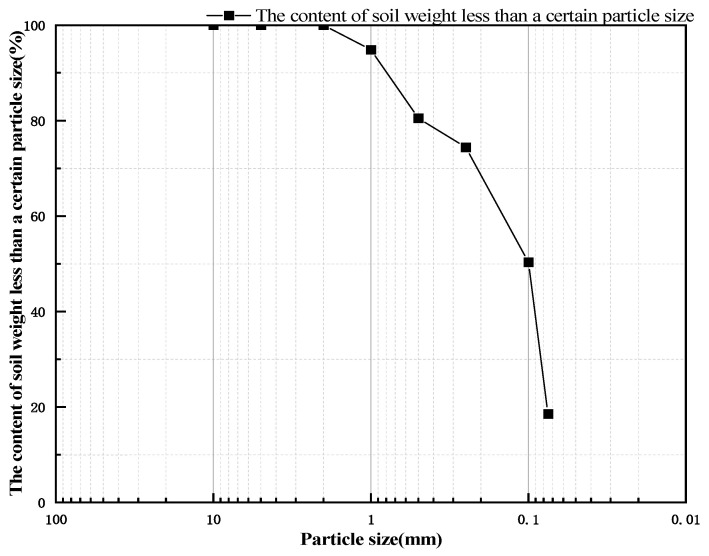
Particle size distribution curve of the test soil sample.

Soil samples were collected from a slope in Xuan Hua District, Zhangjiakou City, with a sampling depth of 0.5 m. After collecting, the soil samples were dried, crushed, and sieved through a 2 mm sieve. The soil samples were then sieved according to the “Standard for Soil Testing Methods” (GB/T50123-2019) [18] to obtain the particle size distribution curve shown in Figure 1. The plastic limit of the soil sample is 16.9%, the liquid limit is 27%, and the plasticity index is 10.1. According to the “Code for Design of Building Foundations” (GB/5007-2011) [19], the soil is classified as silty clay. Through compaction tests, the maximum dry density was determined to be 1.85 g/cm^3^, and the optimum moisture content was determined to be 18.1%.

#### 2.1.2. Guar Gum

**Figure 2:**
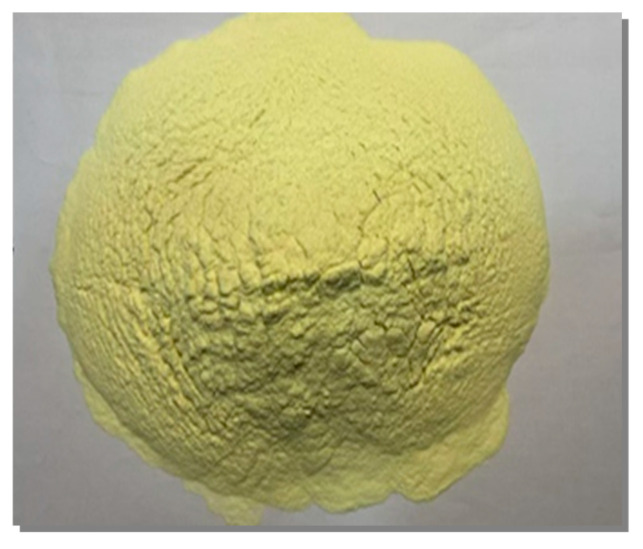
Biopolymer guar gum.

This experiment used industrial-grade guar gum produced by Yufan Biotechnology Co., Ltd. in Zhengzhou, Henan Province, China. Guar gum is a natural high-molecular-weight polysaccharide extracted from the endosperm of guar bean seeds, appearing as light yellow to white powder particles (as shown in Figure 2) with non-toxic and odorless properties [20]. Its main technical specifications are as follows: viscosity (1% aqueous solution at 25 °C) ≥ 5000 MPa·S, purity of 95%, particle size of 80 mesh, and principal components include galactomannan (75–85%), water (8–14%), protein (5–6%), and crude fiber (2–3%). PH value (1% aqueous solution) ranges from 4.0 to 10.5, exhibiting good stability, and it has excellent water solubility [21]. Compared to food-grade guar gum (purity 99%, viscosity 1500 MPa·S), the industrial-grade guar gum used in this experiment differs in purity and viscosity.

### 2.2. Sample Preparation and Process

According to previous research results [22], guar gum was added at concentrations of 0.5%, 1%, 1.5%, and 2% (by weight of guar gum powder to dry soil), with a control group of plain soil. Each group of samples was prepared using the “dry mixing method”, with a moisture content of 18.1% and a compaction degree of 85%. The cylindrical stainless-steel container used in the test had a diameter of 250 mm and a height of 50 mm. To reduce test errors caused by boundary friction, the inner wall was coated evenly with a layer of Vaseline beforehand. The total height of the sample is 30 mm, compacted in two stages, with each stage having a height of 15 mm.

To observe the development of cracks on the soil surface, a dehumidification process was conducted on the test sample. Saturation process: To bring the sample to a saturated state, the saturated moisture content was calculated as 26.83% based on the sample’s known dry density and the soil particles’ relative density. The required amount of water to be added was then calculated as 222 g based on the cylinder volume and the desired saturated moisture content. Water was added in several batches to ensure that the soil particles, water, and guar gum were evenly mixed within the soil. After adding water, the sample underwent slight expansion and deformation. To reduce errors, the sample was covered with plastic wrap, and a lid was placed on top after each addition of water. After adding water to ensure uniform moisture distribution within the soil, the samples were left to stand for 24 h. The moisture content of each sample (with admixture ratios of 0%, 0.5%, 1%, 1.5%, and 2%) was measured by the weighing method, yielding values of 24.36%, 24.68%, 24.89%, 25.01%, and 25.16%, respectively. Thus, the above moisture content differs from the saturated moisture content by 1.67% to 2.47%, which meets the requirements.

Dehumidification process: After the samples reached saturation moisture content, the plastic wrap and lid were removed and allowed to dry naturally. The moisture content was measured at least three times daily using the weighing method based on changes in moisture content. After three days of measurement, the dehumidification process is considered complete if the moisture content changes by less than 0.1% for three consecutive days.

Photography process: The photography equipment used was the following: Manufactured in Shanghai, China SONY (FDR-AX45A), with a resolution of 1440 × 1080. To capture the initial crack conditions of the sample, the first photograph was taken after the sample was saturated. During the dehumidification process, the photography interval was determined based on the cracks’ development and moisture content changes. During the initial stage of the drying process, due to significant crack changes, five photographs were taken daily at four-hour intervals until the fourth day. Subsequently, three photographs were taken daily at six-hour intervals, with the entire drying process continuing for 13 days.

### 2.3. Meteorological Data

**Figure 3:**
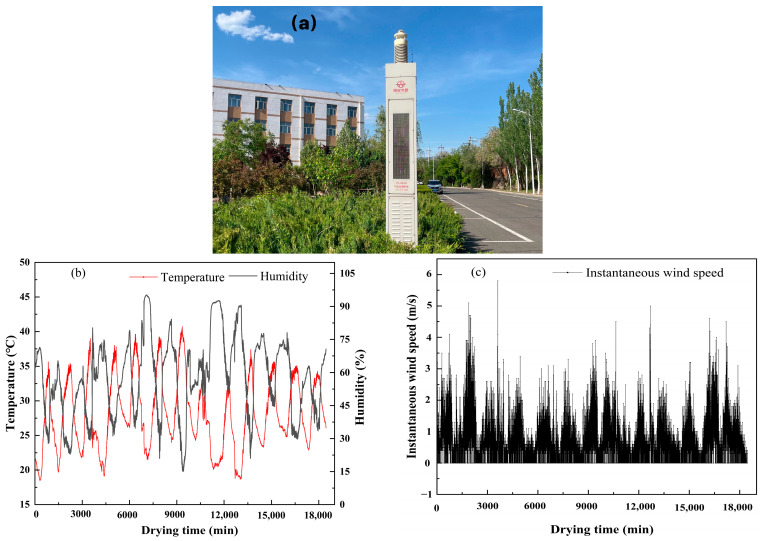
(**a**) Weather monitoring system; (**b**) graph showing changes in temperature and humidity over drying time; (**c**) graph showing wind speed changes over drying time.

An outdoor test area with sunshades was selected for this study to avoid the effects of rainfall on the test specimens. The sunshade in this area also provides rain protection. When necessary, other measures should be taken to minimize interference from rainfall. The test area is adjacent to automatic meteorological monitoring equipment (Figure 3a), with a relatively short distance between them. Through analysis of actual measurement data, it was confirmed that the impact of the partition wall on meteorological data collection is negligible.

The meteorological data used in this study were obtained from meteorological monitoring equipment located on the campus of a certain school in Zhangjiakou City, Hebei Province (specific geographical coordinates: longitude 114.896884, latitude 40.760156). The data observation period began on 17 July 2024, and ended on 29 July 2024. All data were automatically collected once per minute by monitoring equipment, recording three meteorological parameters: temperature (unit: °C), humidity (unit: %), and wind speed (unit: m/s). Figure 3b,c show the trends of temperature, relative humidity, and wind speed over time.

### 2.4. Crack Image Processing

In the quantitative analysis of fracture network morphology, digital image processing technology has been widely applied [2,23,24]. To conduct a quantitative analysis of the fractures generated during the experiment, we combined Photoshop 2024, Python 2023 programming processing algorithms, and the PCAS image analysis system to process the images.

**Figure 4:**
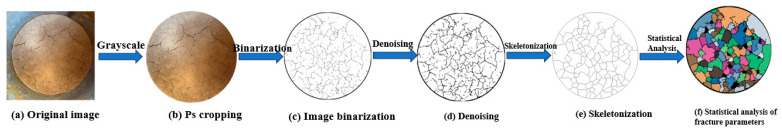
Fissure image processing flowchart.

The image dimensions used in the experiment were 386 pixels × 386 pixels. As shown in Figure 4, taking the final crack image with a guar gum content of 2% as an example, image preprocessing was first performed using Photoshop 2024. Photoshop 2024, as a professional image processing software, was used to separate the sample from its surrounding environment (as shown in Figure 4b). To further eliminate image noise, a custom-written Python programming algorithm was employed for image processing. Python, as a programming language, combined with the OpenCV library, enables automated image processing. The specific steps are as follows: first, the Canny edge detection algorithm was used to accurately identify the crack edges in the image and remove noise (as shown in Figure 4c); then, morphological closing operations were performed using structural elements to connect the edge parts, thereby eliminating isolated minor noise points (as shown in Figure 4d); finally, a connected component analysis was performed to mark the connected regions in the image and set a minimum area threshold to remove smaller connected regions, thereby retaining larger connected regions for further analysis.

Finally, the Pores and Cracks Analysis System (PCAS) was used to extract crack morphology parameters. PCAS is professional image analysis software specifically designed for the field of geotechnical engineering. After processing the images in Python, they were imported into the PCAS system. Following a series of operations, including grayscale value processing, binarization, noise removal, crack network skeletonization (as shown in Figure 4e), and crack block partitioning (as shown in Figure 4f), the cracks were separated, and fissure morphology parameters were obtained, including the number of fissures, average fissure width, total fissure length, total fissure area, surface fissure ratio, and fractal dimension of fissures. The definitions and annotations of these parameters are provided in reference [25].

## 3. Test Results and Analysis

### 3.1. Water Evaporation Process of Specimens

During the sample’s drying and moisture loss process, the evaporation of moisture within the soil causes a change in the sample’s weight. By recording the sample’s weight in real time, it is possible to plot each sample’s average moisture content change curve during evaporation. The formula for calculating the average moisture content of the sample is as follows:

(1) $$\omega_{t}=\frac{m_{\omega }-(m_{0}-m_{t})}{m_{s}}\times 100\%,$$

In the formula, $m_{\omega }$ is the quality of the water used in sample preparation, g; $m_{s}$ is the quality of the soil used in sample preparation, g; $m_{0}$ is the quality of the sample preparation upon completion, g; $m_{t}$ is the sample mass measured at time t, g.

**Figure 5:**
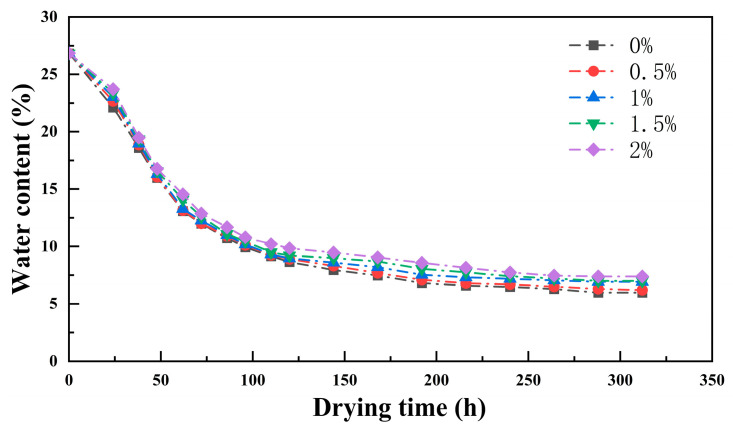
Curves showing changes in guar gum content with drying time.

As shown in Figure 5, the curve represents the relationship between the average moisture content of the sample and drying time. When the average moisture content of the sample reaches 26.83%, it is considered the initial moisture content for the evaporation loss curves of all samples. During the initial stage (≈26.83% to 23%), the evaporation rates of the five samples were relatively low, with sufficient moisture and no cracks. The surfaces of the samples in contact with air remained relatively stable, and the process of water loss through evaporation was also relatively smooth. When moisture drops below 23%, the samples crack as the guar gum content increases. The curves for the five groups with different guar gum contents all show that the rate of water loss accelerates, but the slope of the curves remains stable. When the moisture content dropped below 10%, all samples gradually entered the difficult evaporation stage, with the overall curve showing an upward trend and eventually stabilizing. It can be observed that the average moisture content of the samples decreases with increasing dosage.

### 3.2. Dynamic Evolution Process of Cracks

**Figure 6:**
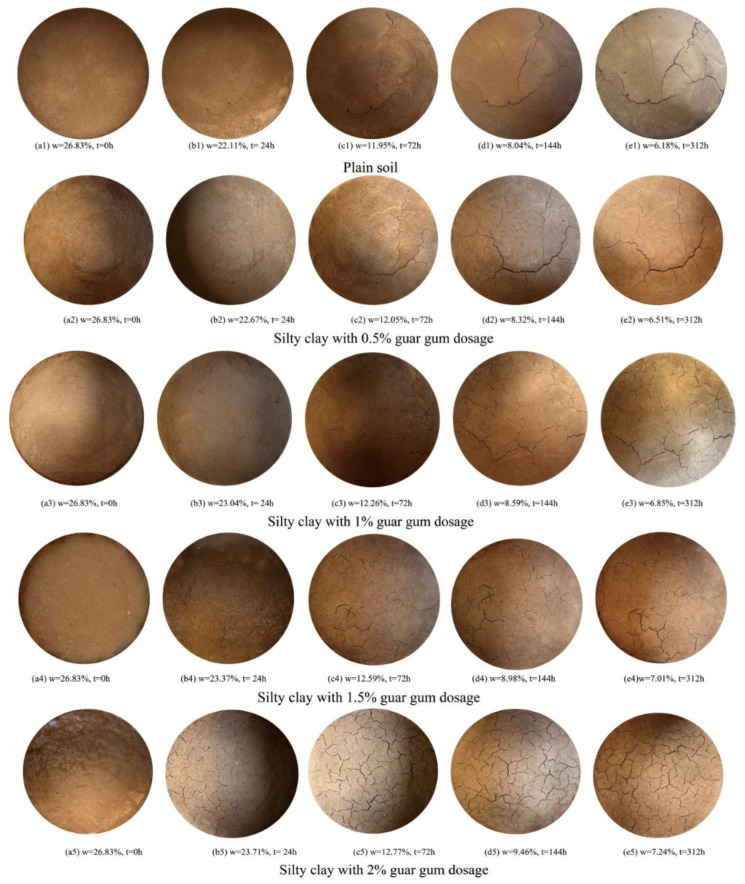
Images of crack development at different guar gum concentrations.

Figure 6 shows the crack development of each sample under different guar gum content conditions during the drying process. By comparing the images of the cracks taken before and after, it is possible to observe the differences in crack morphology under different guar gum contents. It can be observed that when no guar gum is added (as shown in Figure 6(e1)), the soil cracks are visible, and the separated soil blocks are relatively large. As can be seen from Figure 6(e2–e5), with increasing guar gum content, the development of cracks is promoted by the addition of guar gum, leading to an increase in the number of cracks and thereby reducing the formation of large soil clumps. This indicates that guar gum can effectively inhibit the formation of large soil clumps to a certain extent.

### 3.3. Variation Laws of Basic Crack Indicators

**Figure 7:**
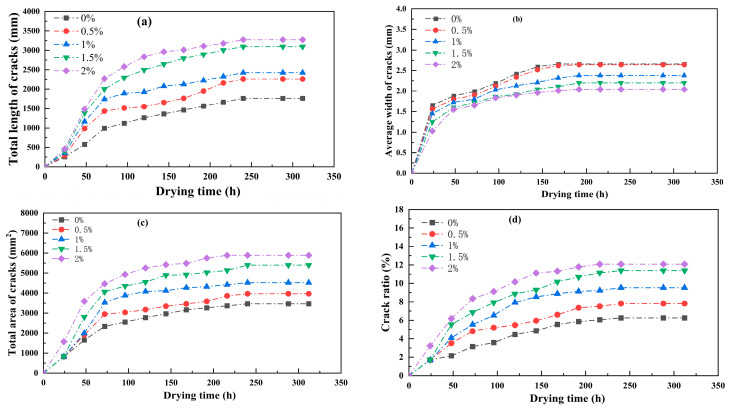
Changes in fissure indicators. (**a**) Graph showing the change in total crack length with drying time; (**b**) Graph showing the change in average crack width with drying time; (**c**) Graph showing the change in total crack area with drying time; (**d**) Graph showing the change in crack rate with drying time.

Image analysis was performed on the crack images of the specimens during the cracking process, and relevant crack parameters were extracted, including the total length of the cracks, the average width of the cracks, the total area of the cracks, and the surface crack rate. The curves showing the changes in these basic parameters with drying time are shown in Figure 7.

As shown in Figure 7a, the total length of the cracks effectively reflects the degree of soil fragmentation. The total crack length exhibits a trend of significant initial increase followed by stabilization. When the guar gum content is 2%, the total crack length increases significantly to 3263.67 mm, while the sample without guar gum content is only 1463.26 mm. The other three samples with different guar gum contents fall between these two values. As the guar gum content increases, the total crack length of the samples gradually increases.

Additionally, the time required for the total crack length to reach a stable state decreases with increasing guar gum content. Furthermore, a longer total crack length leads to an increase in the number of soil block fragments. The fundamental reason for guar gum’s ability to increase the total crack length lies in its unique physical and chemical properties, which alter the stress distribution and water migration patterns within the soil matrix. As a high-molecular-weight polymer, guar gum forms a three-dimensional network structure with active groups on the soil particle surfaces through hydrogen bonding and electrostatic interactions. This structure disperses the few weak areas of the soil during drying and contraction over a larger area, thereby inducing the formation of more microcracks.

The average width of cracks in powdery clay is one of the important factors affecting slope stability. Existing research indicates [26] that, within a specific range, the wider the crack width, the faster the rainwater infiltration rate, and the more likely it is to cause slope instability. As shown in Figure 7b, when no guar gum was added to the soil and when samples with different guar gum contents were added, the average crack widths were 2.66 mm, 2.64 mm, 2.38 mm, 2.22 mm, and 2.02 mm, respectively. The average crack width decreased by 8.27%, 9.8%, 6.7%, and 9%, respectively. These values generally decrease with increasing guar gum content, showing a negative correlation with guar gum content. During drying, samples without guar gum exhibited a continuous upward trend until reaching a stable state. In contrast, samples with different guar gum contents showed a rapid increase followed by a gradual stabilization in the average crack width. Under different guar gum addition ratios, the average crack width of the soil after guar gum addition was lower than that of the unmodified soil without guar gum addition. This indicates that adding guar gum can suppress the average crack width of the soil, and the higher the guar gum addition ratio, the smaller the average crack width. In addition, the decrease in the average width of cracks reflects the reduction in the degree of sample opening, further proving the effect of guar gum on improving the modified soil.

In addition, guar gum has excellent hydrophilic properties, enabling it to adsorb and retain moisture, thereby slowing down the drying rate of soil and reducing internal drying shrinkage. Its binding action also enhances the cohesive force between soil particles, effectively limiting the opening of cracks, ultimately resulting in a decrease in crack width as the guar gum content increases.

At the same time, the total area of cracks can also indicate soil fragmentation. When the guar gum content is 0.5%, the soil cracks are not fully developed, the soil clumps are relatively intact, and the total area of cracks is also relatively small (Figure 7c). As shown in Figure 7d, the fracture rate can reflect the development of the fracture network. Between a water content of 23% and 10%, the fracture rate of the five samples exhibited a nearly linear increase trend; when the water content decreased below 10%, the fracture development of the samples stabilized, with surface fracture rates of 5.26%, 8.14%, 10.53%, 12.02%, and 12.08%, respectively. It can be seen that as the guar gum content increases, the crack rate on the soil surface gradually rises. In summary, adding different amounts of guar gum to the test samples increased the total length of cracks, the total area, and the surface crack rate while effectively reducing the average width of cracks. This narrowed the average width of cracks, slowed down rainwater infiltration, and helped reduce slope instability. This also demonstrates that guar gum can inhibit crack development and control the average crack width within a relatively small range.

### 3.4. Fractal Dimension of Cracks

Fractal dimensions are an important concept for describing natural shapes, contours, or forms. They do not consider complexity and are a key parameter for the fracture morphology of soil [27]. In image analysis, the fractal dimension ranges from [1, 2.0), representing the degree of heterogeneity, with higher values indicating stronger heterogeneity. Typically, the fractal dimension provides the self-similarity or the rate of change in length (perimeter) for measurement (area). Specifically, for a porous system exhibiting fractal characteristics, the relationship between area (S), perimeter (C), and fractal dimension ($D_{f}$) can be expressed as follows:

(2) $$\log(C)=\frac{D_{f}}{2}\cdot \log(S)+C_{1},$$

In the formula, $C_{1}$ is a constant; $D_{f}$ is the fractal dimension; $\frac{D_{f}}{2}$ is the slope of the approximate line. The shape factor represents the roughness of the pore edges $f_{f}=\frac{4\pi S}{C^{2}}$.

Substituting into the equation, we obtain the following fractal dimension:

(3) $$\log(f_{f})=(1-D_{f})\cdot \log(S)-2C_{1}+\log(4\pi ),$$

The fractal dimension can be written as follows:

(4) $$f_{f}=4\pi \cdot 10^{-2C_{1}}\cdot S^{1-D_{F}},$$

#### 3.4.1. The Variation Law of the Fractal Dimension of the Crack Area

**Figure 8:**
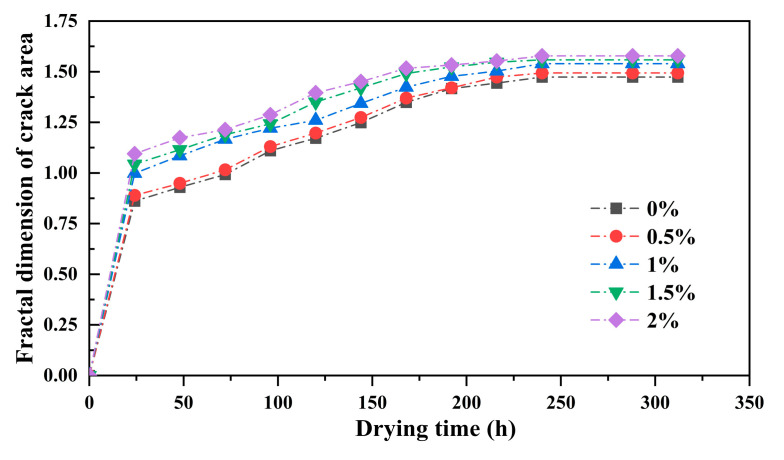
Fractal dimension of crack area as a function of drying time.

The fractal dimension of the crack area is directly calculated after the binarization of the crack image. Figure 8 shows the relationship curves between the fractal dimension of the crack area and the drying time for each sample.

As the drying time increases, the fractal dimension of the crack area gradually increases. Between 0 and 24 h, the fractal dimension of the crack area shows a rapid increase; between 24 and 200 h, as the crack network further develops, the fractal dimension continues to increase; between 200 and 312 h, the fractal dimension stabilizes and no longer shows significant changes. In summary, the change in the fractal dimension of the crack area during drying exhibits distinct stage-like evolutionary characteristics, namely rapid growth followed by sustained development and, ultimately, stabilization. Ultimately, the fractal dimension stabilizes within the range of 1.55–1.65, and the curve of its variation with drying time is largely overlapping.

This indicates the dynamic process of crack development during drying under guar gum modification and further suggests that the addition of guar gum has a relatively minor influence on the fractal dimension of the crack area, confirming the limitations of its impact on the final morphological characteristics of drying cracks.

#### 3.4.2. The Variation Law of the Fractal Dimension of Crack Length

There is a significant difference between the fractal dimension of the length of the fissure and the fractal dimension of the area. When calculating the fractal dimension of the length of a fracture, the fracture is extracted, binarized, and skeletonized. Finally, the fractal dimension of the fracture skeleton is statistically analyzed and calculated. The advantage of this method lies in ignoring the influence of the fracture area and focusing solely on fracture length and its planar distribution characteristics, providing a new perspective for the quantitative assessment of fracture morphology [28].

**Figure 9:**
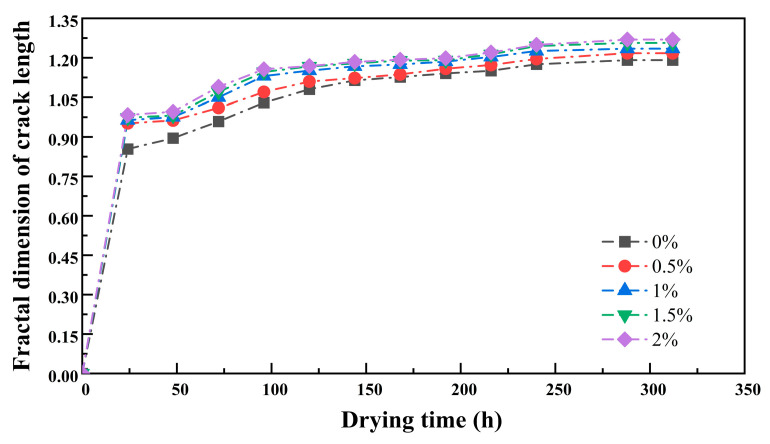
Fractal dimension of crack length as a function of drying time.

Figure 9 shows the relationship curve between the fractal dimension of the crack length and the drying time. Under different guar gum content conditions, the surfaces of the samples with added guar gum exhibited dense cracks, with the crack frameworks forming a network-like structural morphology. Consequently, the corresponding crack length fractal dimensions were relatively high, at 1.217, 1.236, 1.255, and 1.269, respectively. In contrast, the surfaces of the samples without added guar gum were relatively simple, primarily consisting of larger cracks and some minor corner cracks, with a crack length fractal dimension of only 1.201. Within 0–24 h, the fractal dimension of crack length increased rapidly; between 24 and 200 h, the trend of the fractal dimension of crack length was consistent with that of the fractal dimension of the crack area; between 200 and 312 h, with increasing guar gum content, the curves showed some differences, and the fractal dimension of crack length slightly increased. This indicates that it can effectively reflect cracks’ morphology and distribution characteristics to a certain extent.

The fractal dimension of the length of the fissures shows a trend that is generally consistent with that of the fractal dimension of the area of the fissures. However, the numerical values of the length fractal dimension are generally lower than those of the corresponding area fractal dimension. This discrepancy is primarily due to the neglect of the influence of the fissures on the fractal dimension, indicating that the area of the fissures does indeed have a significant impact on the fractal dimension.

The rate of fissure generation is an important indicator of the speed of fissure development. The total area of fractures can effectively measure the development rate within a unit of time, and the total length of fractures.

(5) $$V_{S}=\frac{S_{t}}{t},$$

(6) $$V_{L}=\frac{L_{t}}{t},$$

In the formula, $V_{S}$ represents the development rate of the total fracture area; $S_{t}$ denotes the total fracture area corresponding to time t; $V_{L}$ represents the development rate of the total fracture length; $L_{t}$ denotes the total fracture length corresponding to time t.

**Figure 10:**
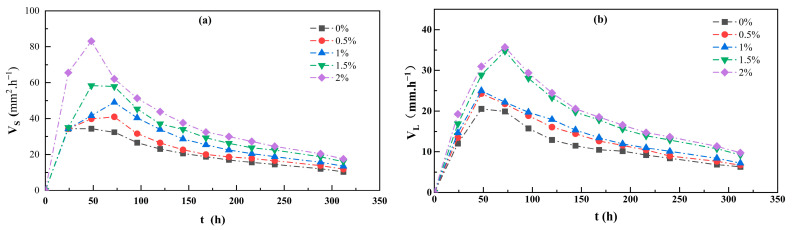
Relationship between crack development rate and drying time. (**a**) Figure showing the rate of development of total crack area as a function of drying time; (**b**) figure showing the rate of development of total crack length as a function of drying time.

As can be seen from Figure 10, both the total area development rate and the total length development rate of cracks first increase rapidly to a peak and then slowly decrease to a stable state as the drying time decreases. Within 0–24 h, cracks are in their initial development stage. For samples with different guar gum contents, the total area development rate and total length development rate of cracks are similar, and both reach their peak values. After 24 h, cracks enter a rapid development stage. As the guar gum content increases, the total area development rate and total length development rate of cracks show a decreasing trend.

In summary, the development rates of the total area and total length of cracks during the drying process exhibited significant stage-dependent differences under different periods and guar gum addition levels. This clearly reveals the mechanism by which guar gum inhibits crack propagation and provides a dynamic evolutionary perspective on the role of guar gum addition during the drying process.

**Figure 11:**
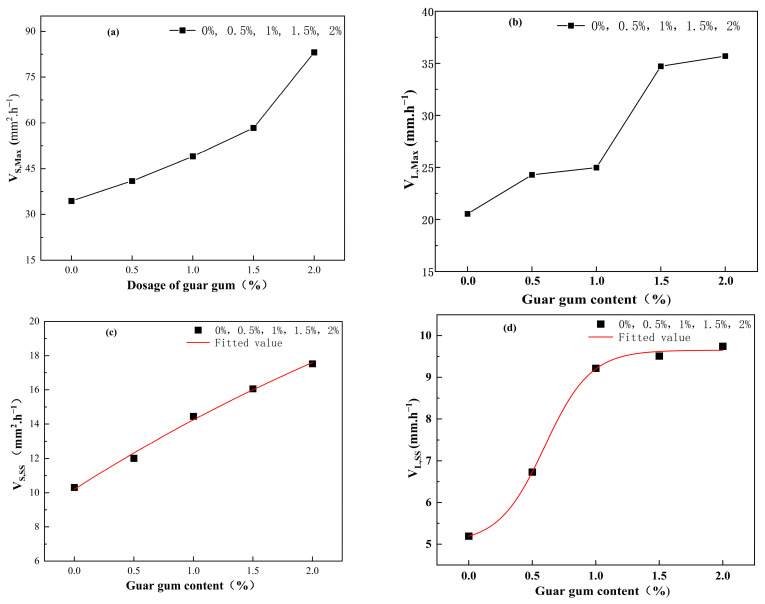
Fracture development rate. (**a**) Peak development rate of total crack area; (**b**) Peak development rate of total crack length; (**c**) Stable development rate of total crack area; (**d**) Stable development rate of total crack length.

As the guar gum content increases, the peak development rate of the total crack area (V_S,Max_) shows an upward trend, while the peak development rate of the total crack length (V_L,Max_) exhibits an “M”-shaped trend (see Figure 11a,b). Similarly, as the guar gum content increases, the stabilization rates of the total crack area (V_S,SS_) and total length (V_L,SS_) also increase (see Figure 11c,d). Additionally, the stabilization rates of the total crack area and total length were fitted with the guar gum content, showing a high degree of agreement. Therefore, this fitting has important reference value in related studies.

### 3.5. Probability Entropy of Cracks

Probability entropy is an important indicator describing the directionality of fracture expansion [29]. Its expression is as follows:

(7) $$H=-\sum_{i=1}^{n}P_{i}\log_{n}P_{i},$$

In the formula, *H* is the probability entropy; $P_{i}$ represents the percentage of fractures within a specific range; *i* represents the number of the division direction range; n represents the number of division direction ranges. For example, when i = 1, the direction range of the crack is 0–10°; n indicates that the direction range of 0–180° is divided into 18 parts, i.e., n = 18. The value of probability entropy H ranges from 0 to 1; when H = 0, it indicates that the fracture directions are entirely consistent; when H = 1, it means that the fracture distribution is randomly oriented, and the larger the value of H, the more the fracture expansion direction tends to be random. As the probability entropy increases, the cracks become increasingly chaotic.

**Figure 12:**
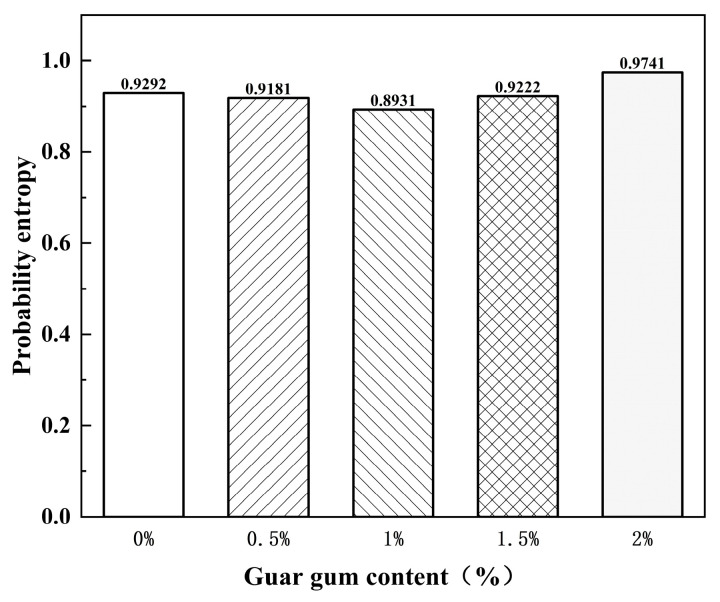
Guar gum content and probability entropy.

In the five sets of samples, since the probability entropy values measured each time were relatively close and the variation was less than 3%, it can be concluded that the guar gum content has a minor effect on the probability entropy of cracks in powdery clay bodies. To reduce experimental errors, the probability entropy of the samples was taken as the average value of different guar gum contents. As can be seen from Figure 12, the addition of different guar gum contents does not significantly affect the cracking direction of powdery clay. As the guar gum content increased, the probability entropy value fluctuated slightly, and the crack expansion of the samples showed a trend of first decreasing and then increasing.

By comparing the final cracking images of plain soil specimens without guar gum addition and specimens with guar gum addition, it can be observed that as entropy increases, the initial expansion direction of cracks becomes approximately completely random, consistent with the variation pattern of probability entropy.

## 4. Analysis and Discussion

### 4.1. Water Loss Process

The samples’ evaporation and moisture loss process increase in duration with the addition of guar gum, primarily due to the varying guar gum content in each sample. This results in differing rates of moisture loss within the same time frame. Theoretically, this is due to an increase in guar gum content, which increases the adhesive force of the matrix, making water migration more difficult and keeping the evaporation rate stable over a longer time. This phenomenon can be attributed to the “thickening” effect [30].

Secondly, the higher the guar gum content, the earlier the soil enters the difficult evaporation stage. In contrast, at lower guar gum contents, the soil shows little or no significant difficulty in evaporation. From the perspective of the “thickening” effect, when the guar gum content is low, the total length, total area, and crack rate of the cracks are relatively short. In contrast, the average width of the cracks is wider, resulting in larger soil clumps formed by segmentation. This prolongs the water migration path, weakens capillary action, and slows the evaporation rate. Conversely, as the guar gum content increases, the cracks’ total length, total area, and crack ratio increase relatively, resulting in smaller soil blocks formed by segmentation. The average width of the cracks narrows, shortening the water migration pathways and reducing migration difficulty. Therefore, the “thickening” effect can reasonably explain the changes in evaporation rates exhibited by soils with different guar gum concentrations during water loss.

### 4.2. Cracking and Fracture Evolution

**Figure 13:**
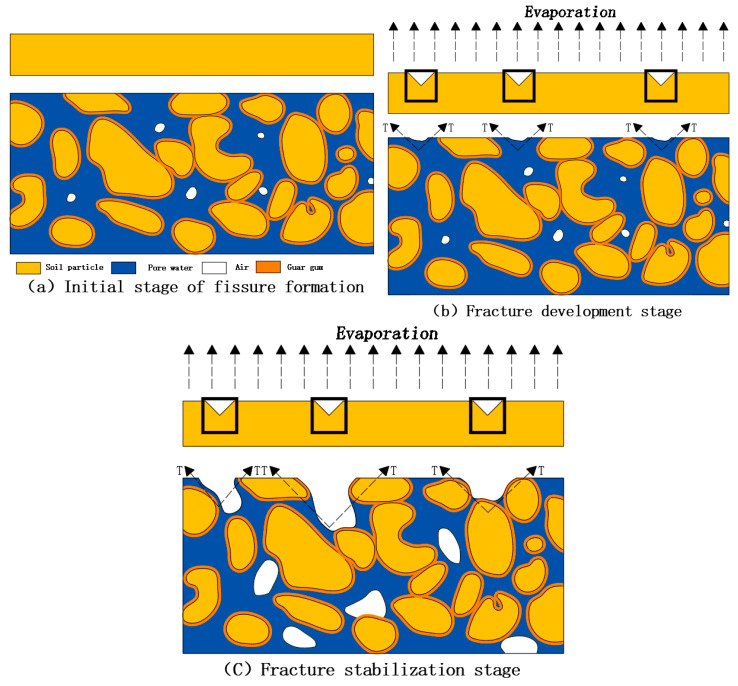
Mechanism of guar gum-modified soil crack evolution.

The experiment investigated the effect of different guar gum contents on soil crack formation under identical outdoor environmental conditions. Figure 13 illustrates the evolutionary mechanism of guar gum-modified soil cracks. The modified guar gum soil underwent three stages: initial crack formation (Figure 13a), crack initiation and expansion (Figure 13b), and crack stabilization (Figure 13c).

Fractures are a special form of soil failure, significantly different from the shear failure caused by external forces under normal conditions. They are tensile failures caused by internal forces resulting from shrinkage due to water evaporation [31,32,33]. During the initial stage of crack formation, the specimen is in a saturated state, with pores filled with water and guar gum randomly distributed throughout the specimen (as shown in Figure 13a). During the drying test, moisture on the sample’s surface gradually evaporates into the air, causing the moisture content of the surface layer to decrease continuously. At the same time, due to capillary action, moisture from the lower layers continuously migrates upward, accelerating the drying of the surface layer. During this process, soil particles undergo displacement and rearrangement, and the space originally occupied by pore water is gradually filled by soil particles, resulting in macro-level shrinkage and deformation of the soil.

During the crack development stage, the sample transitions from saturated to non-saturated as moisture evaporates. Due to reduced moisture content and increased matrix suction [34,35], the effective contact area between the soil and guar gum and the normal stress increase as the soil dries, thereby enhancing tensile strength, forming a guar gum–gas–water interface, and generating tensile force (T) [36]. At this point, any surface defects (such as uneven pore size distribution or bubble formation) can serve as entry points for air intrusion, leading to the gradual formation of cracks. The fundamental cause of crack formation is the imbalance of tensile stress (T) from increased absorptive force within the matrix. As the suction force of the matrix increases, the crack surface pushes soil particles out of the invaded pores. Continuous evaporation along the crack surface increases the pore size at the tip, and the crack further expands in directions perpendicular to and away from the air–water interface. The crack releases stress along the path of the least energy, typically forming interconnected polygonal networks. In surface areas where soil particles are weakly connected, tensile stress concentrates, causing the soil to move toward the center of contraction. When the tensile stress outside the soil exceeds or equals the tensile strength of the soil, the connections between particles break, resulting in the formation of cracks [37,38] (as shown in Figure 13b). At this point, guar gum begins to exert its effect, partially inhibiting the development of cracks. Due to the interlocking, friction, and bonding interactions between soil particles and guar gum, the total length of cracks, total crack area, and surface crack ratio increase. However, the average crack width is effectively reduced, thereby helping to mitigate the risk of slope instability.

During the crack stabilization stage, crack formation is a process of local strain energy release, causing the strain energy of the soil to return to equilibrium. The crack is stabilized when the energy release rate and evaporation shrinkage rate reach a dynamic equilibrium. After cracks form, the soil continues to shrink due to ongoing evaporation. In addition, stress concentration at the tips of the cracks causes them to expand further until they eventually reach a stable state (as shown in Figure 13c). Even in the stable stage, there is still a possibility of further expansion of the cracks.

## 5. Conclusions

This study aimed to investigate guar gum’s effect on silty clay’s dry shrinkage. The formation patterns and evolution mechanisms of cracks in silty clay under different guar gum addition levels were analyzed, and the results were compared with those of unmodified soil samples without guar gum addition. The following main conclusions were drawn:

(1) Adding guar gum can improve the water retention capacity of powdery clay. As the amount of guar gum added increases, the average moisture content of the samples shows a gradual decreasing trend. The final samples have a higher average moisture content than the unmodified soil without guar gum.

(2) The addition of guar gum increases the total length of cracks, the total area of cracks, and the surface crack ratio but effectively reduces the average width of cracks, slowing down the rate of rainwater infiltration and helping to reduce slope instability.

(3) The fractal dimension of the crack area and the fractal dimension of the crack length increase gradually with increasing drying time. With changes in drying time, the total length and area of cracks exhibit a trend of first increasing to a peak and then slowly decreasing. The stabilization rates of total crack length and total area correlate well with guar gum content, making this study of significant reference value.

(4) The addition of guar gum reveals that the larger the entropy, the more random the initial expansion direction of the cracks, which is consistent with the variation law of probability entropy.

## Data Availability

The original contributions presented in this study are included in the article. Further inquiries can be directed to the corresponding authors.
